# Morning versus Nocturnal Heart Rate and Heart Rate Variability Responses to Intensified Training in Recreational Runners

**DOI:** 10.1186/s40798-024-00779-5

**Published:** 2024-11-06

**Authors:** Olli-Pekka Nuuttila, Heikki Kyröläinen, Veli-Pekka Kokkonen, Arja Uusitalo

**Affiliations:** 1 Faculty of Sport and Health Sciences, University of Jyväskylä, P.O. Box 35 (VIV), Jyväskylä, FIN-40014 Finland; 2 UKK Institute for Health Promotion Research, Tampere, Finland; 3 Department of Sports and Exercise Medicine, Clinicum, University of Helsinki, Helsinki, Finland; 4 Clinic for Sports and Exercise Medicine, Foundation for Sports and Exercise Medicine, Helsinki, Finland

**Keywords:** Parasympathetic nervous system, Autonomic nervous system, Orthostatic test, Overload, Endurance training

## Abstract

**Background:**

Resting heart rate (HR) and HR variability (HRV) are widely used parameters to assess cardiac autonomic nervous system function noninvasively. While resting assessments can be performed during sleep or after awakening, it would be relevant to know how interchangeable the results of these measurements are. This study aimed at examining the alignment between nocturnal and morning assessments during regular endurance training and in response to intensive training. A total of 24 recreational runners performed a 3-week baseline period (BL) and a 2-week overload (OL) period (Lucia’s training impulse + 80%). Their running performance was assessed with a 3000-m running test after the BL and OL. The participants recorded daily their nocturnal HR and HRV (the natural logarithm of the root mean square of successive differences; LnRMSSD) with a photoplethysmography-based wrist device and performed an orthostatic test (2-min supine, 2-min standing) every morning with a chest-strap HR sensor. The HR and LnRMSSD segments that were analyzed from the nocturnal recordings included start value (SleepStart), end value (SleepEnd), first 4-h segment 30 min after detected sleep onset (Sleep4h), and full sleep time (SleepFull). The morning segments consisted of the last-minute average in both body positions. All segments were compared at BL and in response to the 3000-m test and OL.

**Results:**

All nocturnal HR and LnRMSSD segments correlated with supine and standing segments at BL (*r* = 0.42 to 0.91, *p* < 0.05). After the 3000-m test, the HR increased and LnRMSSD decreased only in the SleepStart, Sleep4h, and SleepFull segments (*p* < 0.05). In response to the OL, the standing HR decreased (*p* < 0.01), while the LnRMSSD increased (*p* < 0.05) in all segments except for SleepStart. The Pearson correlations between relative changes in nocturnal and morning segments were − 0.11 to 0.72 (3000-m) and − 0.25 to 0.79 (OL). The OL response in Sleep4h HR and LnRMSSD correlated with the relative change in 3000-m time (*r* = 0.63, *p* = 0.001 and *r*=-0.50, *p* = 0.013, respectively).

**Conclusions:**

Nocturnal and morning HR and LnRMSSD correlated moderately or highly in the majority of cases during the BL, but their responses to intensive training were not similarly aligned, especially in LnRMSSD. The nocturnal segments seemed to be sensitive to physical loading, and their responses were associated with the performance-related training responses.

**Supplementary Information:**

The online version contains supplementary material available at 10.1186/s40798-024-00779-5.

## Key Points

• Moderate-to-high correlations were found between nocturnal and morning assessments of HR and HRV during regular endurance training in recreational runners.

• In their responses to maximum exercise, nocturnal and morning HR and HRV were not necessarily aligned, as nocturnal segments seemed more responsive to such a stimulus.

• While lower HR and higher HRV were associated with better endurance performance in all recording segments, changes in these parameters after the overload period correlated only in nocturnal segments.

**Supplementary Information:**

The online version contains supplementary material available at 10.1186/s40798-024-00779-5.

## Background

Wearable technology has been one of the leading worldwide fitness trends for some time [1], which can be seen in continuously emerging new innovations to monitor health- and fitness-related biomarkers. A widely applied example in the field of sports and exercise science is the monitoring of heart rate (HR) and HR variability (HRV) that can nowadays be recorded with chest-belts [2], electrocardiography-based sensors [3], as well as photoplethysmography (PPG)-based watches [4], rings [5], and phone cameras [6]. Although PPG-based recordings are actually measuring pulse rate and its variability (PRV), and it can be argued that PRV and HRV are not exactly interchangeable [7], the PPG-based results align well with the results of HR and HRV, at least in resting condition [4–6, 8]. Daytime HR [9] and HRV [10] can be used to monitor the internal intensity of an activity or stress-recovery responses to different tasks [3, 11], while at rest, HRV is primarily used to assess the current state of recovery and readiness to train/perform [12, 13].

The physiological rationale of HRV is to assess functions of the autonomic nervous system noninvasively, and in particular its parasympathetic branch modulation [14]. The phenomenon has been demonstrated by blocking the vagally-mediated parasympathetic modulation to the heart pharmacologically, which has led to diminished HRV [15, 16]. The same phenomenon can also be observed during exercise, when the parasympathetic activity decreases, and the HRV reaches its minimal values after a certain intensity threshold [10]. The decrease in HRV at higher HR values is also promoted by the nonlinear relationship between RR interval and HR, which means that the same changes in HR cause much greater fluctuations of RR intervals for the slow average HR than for the fast average HR [17]. In general, parasympathetic activity and HRV are at their highest while resting during sleep [18]. To minimize external disturbances and to assess parasympathetic function reliably, resting HRV recordings are typically performed either during sleep or immediately after awakening in supine, sitting, or standing positions [12].

The purpose of the HRV monitoring in sports context is to assess individual’s internal response to training [19, 20]. In theory, high activity of the parasympathetic nervous system at rest, and thus high HRV, reflects a balanced state of cardiovascular homeostasis and good capability to adapt to training stimulus [13]. Whereas low HRV and diminished activity of the parasympathetic nervous system can relate to ongoing recovery-adaptation processes and a compromised capability to adapt to new stimuli [13]. The simplified view that the higher the HRV, the better the state of recovery has also been challenged, as there are situations during which the “hyperactivity” of the parasympathetic nervous system can indicate functional overreaching [21]. Therefore, a significant deviation from the normal range (of an individual) in either direction is often regarded as an abnormal and undesirable response [22–24]. A proactive approach in HRV-monitoring has been applied in the recent interventions examining HRV-guided endurance training prescription [25]. In essence, the training has been intensive, or it has continued without recovery periods as long as the HRV has been within the normal range, defined individually and based on observed day-to-day variation of HRV (e.g., ± 0.5 x within-individual SD) [22–24]. A meta-analysis of HRV-guided studies reported individually adjusted training to have a medium-sized effect on submaximal physiological parameters and a small-sized effect on maximum performance in comparison to predefined training [25].

Despite the wide range of application opportunities and research interest in HRV, there is still a lack of knowledge regarding the potential impact of assessment timing on the observed responses. There are a few studies [26–28] that have examined both morning and nocturnal HRV during regular training periods, but none of them have reported training adaptations and their possible associations with HRV responses. Daytime [13, 19] and nocturnal [20, 29] HRV responses to standardized heavy exercises have been examined in separate studies, but the alignment between the responses within the same participants has not been reported. A similar limitation concerns responses to overload training and the state of functional overreaching – both morning and nocturnal assessments have been applied [21], but not with the longitudinal settings within the same participants. These comparative viewpoints would be relevant in practice when considering the sensitivity of different methods to detect meaningful differences in the state of recovery.

The purpose of this study was to examine the alignment between morning and nocturnal HR and HRV during regular training in recreational runners. Furthermore, the day-to-day and weekly responses to intensified training were compared against the recording conditions. Finally, the study evaluated explanatory factors behind the responses as well as the associations between the responses and training-induced adaptations.

## Methods

### Participants

A total of 32 (14 female) healthy recreational runners, training regularly 4–6 times per week, were recruited to participate in the study. The participants were recruited through the university’s communication channels and social media groups. Before acceptance, their health status was confirmed via a questionnaire to exclude any diseases or regular medications that could have affected the participation. In addition, their resting electrocardiography was recorded and approved by a physician before the final acceptance. In the current study, only the participants that performed the prescribed baseline and overload periods as planned (6 dropouts) and had sufficient adherence (> 80%) to HRV recordings (2 dropouts) were included in the analyses. Thus, the final sample consisted of 24 participants (10 female) aged from 28 to 48 years. All the participants gave their written consent to participate, and the study protocol was approved by the ethics committee of the University of Jyväskylä.

### Study Design

The overall study period included 3 weeks of baseline training, 2 weeks of overload training, and one week of recovery. More detailed information about the whole intervention protocol has been reported in another publication [30]. During the baseline period, the participants continued their regular training in terms of frequency (4–6 sessions per week) and volume (∼ 3.5–7 h per week). Except for a self-paced field running test performed once a week, the training intensity was limited below the first lactate threshold to ensure sufficient state of recovery before the overload period. During the overload period, the participants increased their training load (Lucia’s training impulse [31]) from the baseline period by 80% via increasing training volume (+ 45%) and the number of sessions between the first and second lactate thresholds (3–4 sessions per week). During the recovery period, the training load decreased by 40% from the baseline period and was again low intensity by nature. Each period was preceded and followed by a 3000-m running test. Nocturnal HR and HRV recordings and morning orthostatic tests were performed daily throughout the study. The chosen setting focused on the HR and HRV data collected during the baseline and overload periods and on the running tests that were performed before and after the overload period.

### Measurements

#### Running Performance

Maximum endurance performance was monitored with a 3000-m running test and a self-paced field running test. The 3000-m running tests were performed in small groups (max. 6 persons) on a 200-m indoor track (*n* = 18) or on a 400-m outdoor track (*n* = 6). The outdoor track was used for some participants (in all tests) due to the summer lockdown of the indoor track that was not known when the timetable of the data collection was designed. A standardized 15-min low-intensity warm-up including 3 × 20-30-s accelerations to the target speed was always performed before the test. Verbal encouragement and split times (1000 m and 2000 m) were given to all participants, and blood lactate (post 2 min) was analyzed after each test. The blood lactate samples were drawn from the fingertip and analyzed with Biosen S_line Lab + lactate analyzer (EKF Diagnostic, Magdeburg, Germany).

The self-paced field running test consisted of a perceived exertion-based 15-min warm-up (6-min 9/20, 6-min 13/20, 3-min 17/20) and a 6 × 3-min/2-min recovery interval session that was performed at maximum sustainable effort. The test was instructed to be performed once a week in the same or similar environment and at the same time of day (± 2 h). The average speed of a similar interval session has previously been strongly associated with the 3000-m running performance [32], and it was considered to be a valid marker of the current maximum endurance capacity.

#### HR and HRV Assessments

**Fig. 1:**
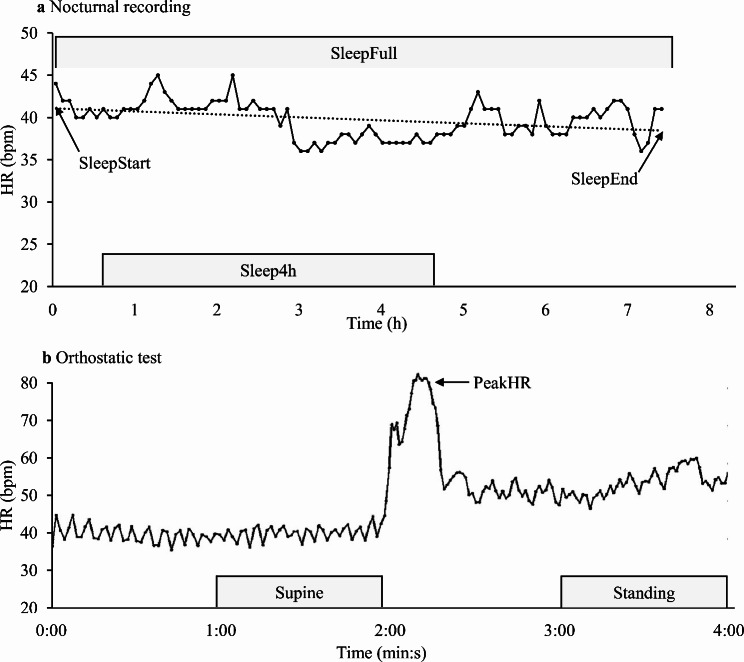
Representation of the segments that were analyzed from the nocturnal recording (**A**) and from the orthostatic test (**B**). HR, heart rate; SleepFull, average of the full sleep time; SleepEnd, end point of linear fit between 5-minute averages of full-night; SleepStart, starting point of linear fit between 5-minute averages of full-night data; Sleep4h, 4-hour period starting 30 min after going to sleep

An orthostatic test was performed every morning in home environment with a chest-belt HR sensor (Polar H10, Polar Electro Oy, Kempele, Finland) that was paired with a watch (Polar Vantage V2, Polar Electro Oy, Kempele, Finland). The validity of the HR-sensor to record RR-intervals has been examined e.g., by Gilgen-Amman et al. [2]. The participants were instructed to perform the test after waking up and emptying their bladder. Before starting the test, they were asked to rest for approximately one minute. The orthostatic test consisted of a two-minute recording in a supine position and a two-minute recording in a standing position after active standing up. The test was performed with the “orthostatic test” feature of the watch, and it informed the participants when it was the right time to change the position and alerted once the recording period was finished. The results that were automatically provided by the watch were the average HR and RMSSD for the last minute in the supine position (Supine), the average HR and RMSSD for the last minute in the standing position (Standing), and the peak HR achieved during the first 30 s of the standing position. (Fig. 1).

The nocturnal HR and RMSSD were recorded with a PPG-based wrist-worn watch (Polar Vantage V2). The validity of the device to measure HR and RMSSD has been reported previously [4]. The participants received advice about the proper attachment of the watch with the instructions provided by the manufacturer. The recording started automatically after the onset of sleep was detected. The accuracy of the sleep detection algorithm has been examined by Pesonen and Kuula [33]. The results were first synchronized with the Polar Flow software (Polar Electro Oy, Kempele, Finland), after which they were automatically transferred to Coach4Pro software (Coach4Pro Oy, Espoo, Finland). The results were provided as consecutive 5-min averages. In the current study, four different types of nocturnal segments were used for the analysis (Fig. 1). The segments were chosen based on previous studies reporting nocturnal assessment results [20, 26].

1. The average of the full sleep time (SleepFull).
2. The 4-hour period starting 30 min after going to sleep (Sleep4h).
3. The starting point of linear fit between 5-minute averages of full-night data (SleepStart).
4. The end point of linear fit between 5-minute averages of full-night data (SleepEnd).

The results that were analyzed similarly from the morning orthostatic tests and nocturnal recording segments included:

1. Baseline: The within-participant average and coefficient of variation (CV) during the 3-week baseline period.
2. Acute response: The night and morning results after the 3000-m running test compared with the preceding night and morning results.
3. Overload response: The 7-day average [21] at the end of the overload period compared with the 3-week baseline period.

All RMSSD results were transformed to natural logarithm form to allow normal distribution for all segments in accordance with the suggestions of Plews et al. [34]. Although log-transformed values were used in the analyses, the absolute values at the baseline are provided as a supplemental file (Additional file 1). RMSSD was used as the only HRV parameter because: (1) It is the parameter that most wearables provide for the user; (2) Practitioners have been encouraged to focus on this particular parameter due to its reliability and applicability in day-to-day monitoring [34] (3) RMSSD has been well validated as a marker of parasympathetic nervous system activity [15, 35]. There were two participants whose data from the night after the 3000-m running test was missing, and therefore only the results of the morning assessment were used to analyze the acute responses of these participants. Raw pulse-to-pulse- or RR-interval data was not available for the recordings; thus, the artifact correction and possible data filtering were based on proprietary algorithms of the watch/software, similar to the actual users of these wearables.

### Statistical Analysis

The results are presented as mean ± standard deviation. The normality of the data was analyzed with the Shapiro-Wilk test. A general linear model repeated measures test was used to compare the baseline results of different recording conditions/segments and to assess their changes in response to maximum exercise or overload period. The time points (baseline, pre-3000-m, post 3000-m, post overload) and analysis segment (Supine, Standing, SleepFull, SleepStart, SleepEnd, Sleep4h) were used as within-subject factors for these analyses. Between-segment differences in the responses were compared with the relative changes from the baseline or pre-3000-m, and the analysis segment was then used as a within-subject factor. A Bonferroni post hoc test was used for all within- and between-group comparisons. The Pearson correlation coefficient was used to examine the associations between the absolute values at the baseline and their relative changes after maximum exercise or overload period. In addition, the Intraclass correlation coefficient (2,1) was analyzed for the absolute values at the baseline, and Partial correlation analyses, adjusted for sex, were performed to examine associations between the relative change of HR or LnRMSSD during the overload period and the relative change in 3000-m running performance followed by the overload period. The 95% confidence intervals of the correlations were analyzed using SPSS (IBM SPSS Statistics v.28 software, SPSS Inc., Chicago, IL) default settings, and Fisher method for the bivariate correlations and bootstrap method for the Partial correlation analyses. The linear model and coefficient of determination were analyzed to assess within-individual relationships between HR and LnRMSSD during the study period. The repeated measures correlation [36] was used to analyze associations between the interval running performance and preceding HR or LnRMSSD results. The correlation was calculated with the R studio (version 4.3.1) according to the software instructions and guidelines provided by Marusich and Bakdash [37]. The magnitude of correlations was interpreted according to Mukaka [38]: 0.00-0.30 = negligible, 0.30–0.50 = low, 0.50–0.70 = moderate, 0.70–0.90 = high. 0.90-1.00 = very high. The analyses were performed with the IBM SPSS Statistics v.28 software (SPSS Inc., Chicago, IL).

## Results

### Comparison Between Morning and Nocturnal Assessments at Baseline

There were no differences in the adherence to nocturnal (43 ± 2 recordings, 95.6 ± 1.9%) and morning (43 ± 2 recordings, 96.4 ± 2.0%) recordings during the whole study period. The absolute results and within-participant CV of each recording segment are presented in Table 1. During the orthostatic test, the HR increased from supine to standing position on average by 21.4 ± 5.8 bpm, while the LnRMSSD decreased by 1.1 ± 0.5 ms. The Peak HR of the test was on average 38.0 ± 7.0 bpm higher than the HR in the supine position. During the night sleep that lasted on average 7.2 ± 0.6 h, the HR decreased on average from SleepStart to SleepEnd by 5.3 ± 2.7 bpm, while the LnRMSSD increased by 0.26 ± 0.19 ms. In the current sample, males had significantly lower HR (mean difference 11.5 bpm) and higher LnRMSSD (mean difference 0.43 ms) in all segments compared to females (*p* < 0.05). In the CV, there were no differences between the sexes.

**Table 1:** Morning and nocturnal heart rate (HR) and LnRMSSD results during the 3-week baseline training period

	Mean baseline (range)	Within participant baseline CV % (range)
**HR (bpm)**		
Morning supine^a^ Morning standing^b^ SleepFull^c^ Sleep4h^d^ SleepStart^e^ SleepEnd^f^	52.8 ± 8.2^be^ (39.4–66.9) 74.2 ± 9.2^acdef^ (55.0-93.6) 53.3 ± 7.3^bef^ (41.6–66.9) 53.3 ± 7.5^bef^ (41.0-68.7) 56.0 ± 7.6^abcdf^ (43.3–71.4) 50.7 ± 7.2^bcde^ (40.1–62.9)	6.8 ± 2.4^b^ (4.1–12.8) 9.7 ± 3.1^acdf^ (5.5–18.1) 6.0 ± 2.1^be^ (3.7–11.3) 6.6 ± 2.2^be^ (3.7–11.3) 8.3 ± 1.6^cdf^ (5.7–11.2) 6.5 ± 2.4^bd^ (3.2–12.9)
**LnRMSSD (ms)**		
Morning supine^a^ Morning standing^b^ SleepFull^c^ Sleep4h^d^ SleepStart^e^ SleepEnd^f^	4.03 ± 0.39^bf^ (3.32–4.69) 2.90 ± 0.44^acdef^ (1.94–3.61) 4.15 ± 0.31^bef^ (3.55–4.72) 4.12 ± 0.32^bef^ (3.50–4.69) 4.00 ± 0.34^bcdf^ (3.33–4.62) 4.26 ± 0.30^abcde^ (3.65–4.80)	8.2 ± 3.9^bcdf^ (3.9–18.4) 16.1 ± 5.9^acdef^ (8.7–33.4) 4.2 ± 1.7^abde^ (1.6–8.2) 4.6 ± 1.8^abce^ (1.9-9.0) 6.3 ± 2.4^bcdf^ (2.9–12.6) 4.4 ± 1.7^abe^ (1.7–8.1)

^abcdef^indicates significant (*p* < 0.05) difference between segments. CV, coefficient of variation; LnRMSSD, the natural logarithm of the root mean square of successive differences; SleepFull, average of the full sleep time; SleepEnd, an end point of linear fit between 5-minute averages of full-night; SleepStart, a starting point of linear fit between 5-minute averages of full-night data; Sleep4h, a 4-hour period starting 30 min after going to sleep

**Fig. 2:**
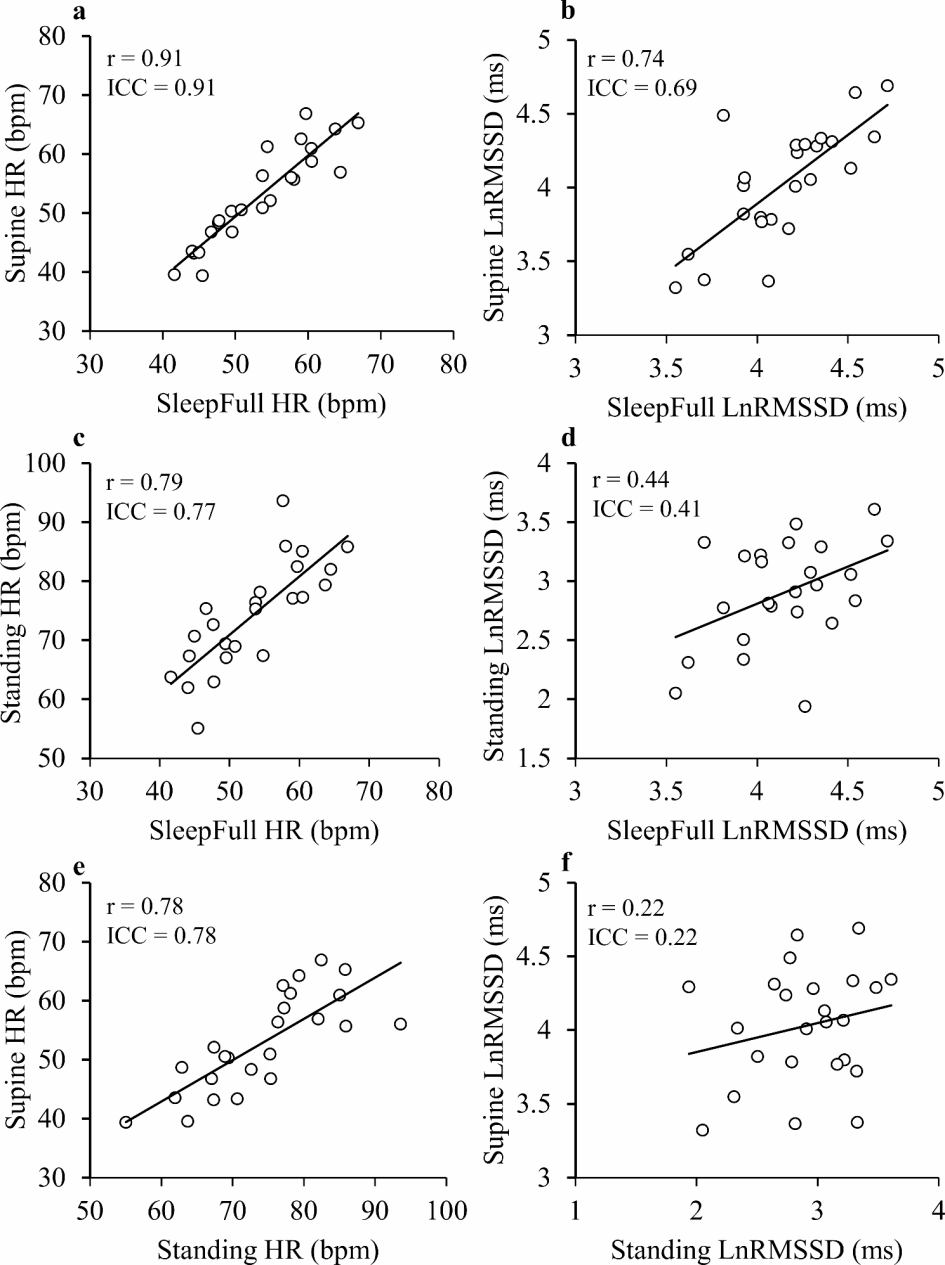
Pearson correlation coefficients between supine, standing, and SleepFull HR and LnRMSSD segments at the baseline. HR, heart rate; LnRMSSD, the natural logarithm of the root mean square of successive differences; SleepFull, average of the full sleep time

The results of HR (*r* > 0.77) and LnRMSSD (*r* > 0.42) segments correlated significantly (*p* < 0.05) with each other at the baseline, except for supine and standing LnRMSSD (*r* = 0.22, *p* = 0.295) (Additional file 1a). Intraclass correlation coefficient for different HR segments varied between 0.74 and 0.99, while for the LnRMSSD segments, it varied between 0.22 and 0.99 (Additional file 2b). Associations between Standing, Supine and SleepFull segments are demonstrated in Fig. 2.

During the whole study period, the within-individual R^2^ for HR and LnRMSSD varied between 0.40 ± 0.26 of Supine segment and 0.65 ± 0.19 of Sleep4h segment. The only significant between-segment difference (*p* < 0.001) was the lower R^2^ of SleepEnd (0.47 ± 0.23) compared to Sleep4h or SleepFull (0.62 ± 0.20). R^2^ for the standing position was 0.47 ± 0.20 and for the SleepStart 0.59 ± 0.20.

### HR and LnRMSSD Responses to 3000-m Running Test

The average time of the 3000-m running test was 12:49 ± 1:50 min: s, and the running time differed significantly (*p* < 0.001) between females (14:07 ± 1:23 min: s) and males (11:53 ± 1:33 min: s). The peak HR was on average 98.0 ± 2.5% of the maximum HR achieved during the incremental treadmill test preceding BL, and the blood lactate after the test was 12.8 ± 3.1 mmol/l.

**Fig. 3:**
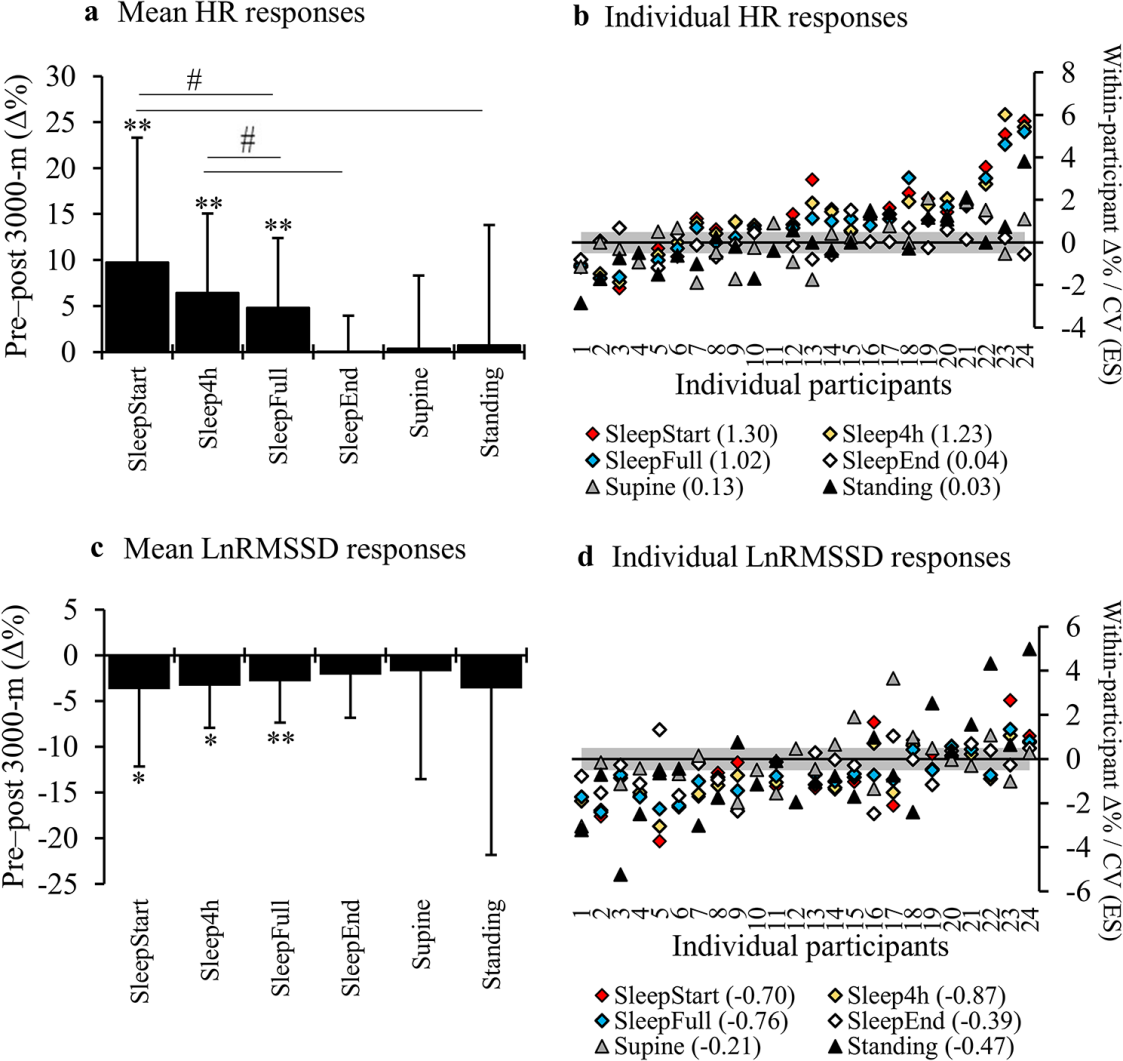
The HR (**A**) and LnRMSSD (**C**) responses from the preceding night or morning to the night or morning after the 3000-m running test. The HR (**B**) and LnRMSSD (**D**) results shown in the brackets are the mean of within participant effect sizes (ES) defined as relative change divided by the within-participant coefficient of variation (CV) during the baseline period. The grey area represents the smallest worthwhile change of 0.5 x CV. *p < 0.05, **p < 0.01 compared to the preceding result. # = significant (*p* < 0.05) between-segment difference in the response. HR, heart rate; LnRMSSD, the natural logarithm of the root mean square of successive differences; SleepFull, average of the full sleep time; SleepEnd, end point of linear fit between 5-minute averages of full-night; SleepStart, starting point of linear fit between 5-minute averages of full-night data; Sleep4h, 4-hour period starting 30 min after going to sleep

Pre-post responses to the 3000-m running test are presented separately for the total group of participants and within individuals in Fig. 3. The detected sleep time (11:23 ± 0:55 PM) started on average 6.1 ± 2.8 h after the 3000-m test, while the orthostatic test (7:13 ± 1:05 AM) was performed on average 14.0 ± 3.1 h after the 3000-m test. The PeakHR of the test, the blood lactate concentration after the test, or the time gap between the recording and the running test were not associated with any of the HR or LnRMSSD responses.

Correlations between the relative changes of the morning and nocturnal HR and LnRMSSD responses are presented in Table 2. Besides the results presented in the Table 2, the relative pre-post 3000-m changes of supine and standing HR (*r* = 0.39, *p* = 0.057) and LnRMSSD (*r* = 0.05, *p* = 0.833) did not correlate significantly. Full correlation analyses including all nocturnal segments are presented in the Additional file 3.

**Table 2:** Pearson correlations coefficients (95% confidence intervals) between the relative changes in the morning and nocturnal segments of heart rate (HR) and LnRMSSD. Correlations are presented separately for the response to 3000-m running test and to 2-week overload period

	Morning supine (pre-post 3000-m Δ%)	Morning standing (pre-post 3000-m Δ%)	Morning supine (BL-OL Δ%)	Morning standing (BL-OL Δ%)
**HR (bpm)**				
SleepFull (Δ%)	0.22 (-0.22;0.59)	0.67*** (0.35;0.85)	0.79*** **(0.57;0.91)**	0.54* **(0.17;0.77)**
Sleep4h (Δ%)	0.18 (-0.27;0.56)	0.68*** **(0.37;0.86)**	0.72*** **(0.44;0.87)**	0.48* **(0.10;0.74)**
SleepStart (Δ%)	0.14 (-0.14;0.64)	0.72*** **(0.42;0.87)**	0.55** **(0.19;0.78)**	0.37 (-0.04;0.68)
SleepEnd (Δ%)	0.35 (-0.09;0.67)	-0.06 (-0.47;0.37)	0.78*** **(0.54;0.90)**	0.52* **(0.14;0.76)**
**LnRMSSD (ms)**				
SleepFull (Δ%)	0.20 (-0.25;0.46)	0.39 (-0.04;0.70)	0.06 (-0.35;0.45)	0.25 (-0.17;0.60)
Sleep4h (Δ%)	0.05 (-0.38;0.46)	0.53* **(0.15;0.78)**	-0.04 (-0.44;0.37)	0.13 (-0.29;0.51)
SleepStart (Δ%)	-0.11 (-0.51;0.33)	0.54* **(0.15;0.78)**	-0.25 (-0.59;0.18)	0.01 (-0.39;0.42)
SleepEnd (Δ%)	0.43* **(0.01;0.72)**	0.03 (-0.39;0.45)	0.31 (-0.11;0.63)	0.45* **(0.06;0.73)**

BL, baseline training period; LnRMSSD, the natural logarithm of the root mean square of successive differences; OL, overload training period; SleepFull, average of the full sleep time; SleepEnd, an end point of linear fit between 5-minute averages of full-night; SleepStart, a starting point of linear fit between 5-minute averages of full-night data; Sleep4h, a 4-hour period starting 30 min after going to sleep. *p < 0.05,**p < 0.01 ***p < 0.001

### HR and LnRMSSD Responses to Overload Period

The weekly training load increased from the baseline period to the overload period as Lucia’s training impulse by 73 ± 11% and as sRPE by 124 ± 46%. The 3000-m running time improved after the overload period (*p* < 0.001) on average by -1.3 ± 1.6% (range from + 2.2% to -4.3%).

**Fig. 4:**
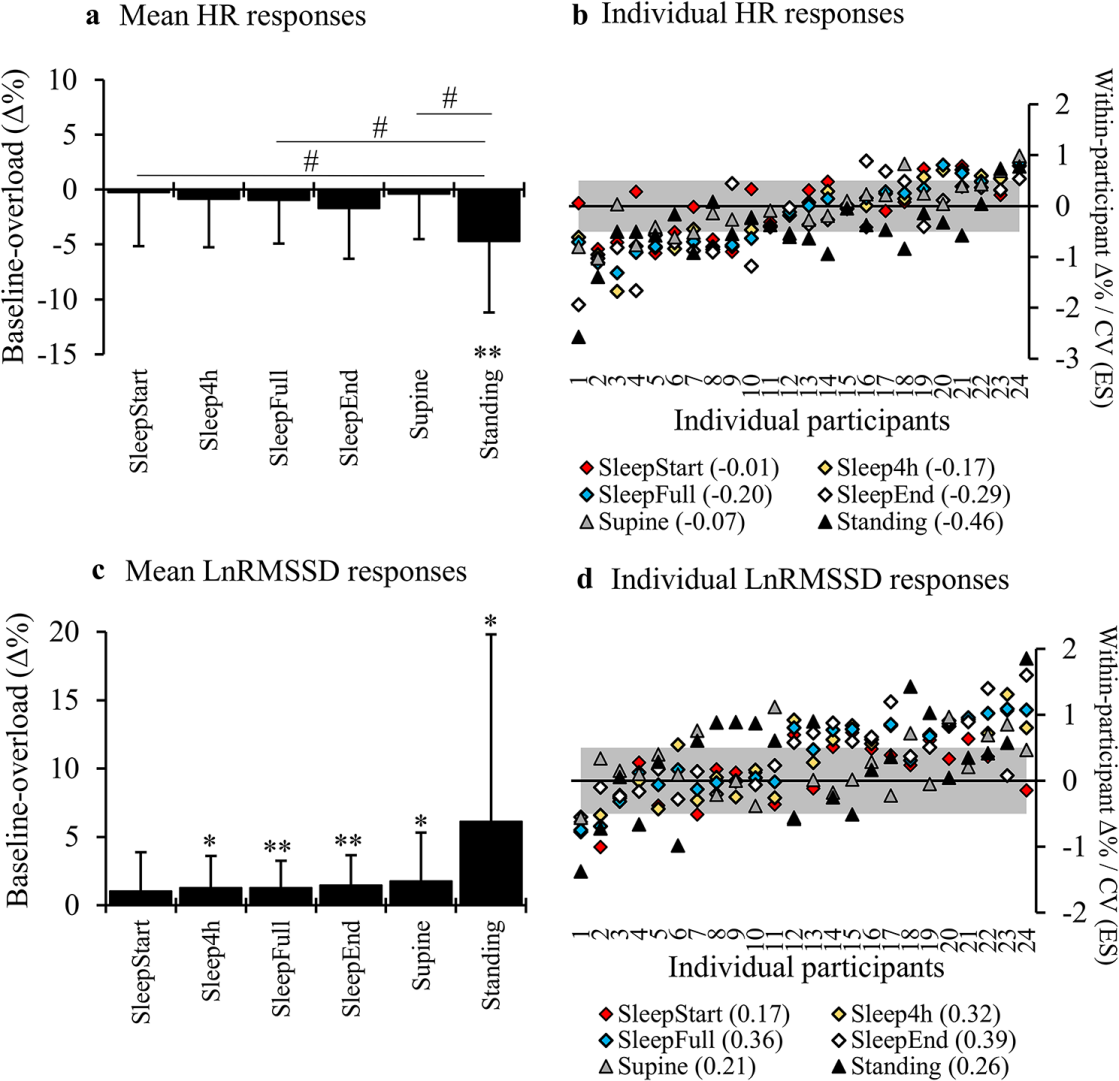
The HR (**A**) and LnRMSSD (**C**) responses from the baseline period to the final week of the overload period in nocturnal and morning segments. The HR (**B**) and LnRMSSD (**D**) results shown in the brackets are the mean of within participant effect sizes (ES) defined as relative change divided by the within-participant coefficient of variation (CV) during the baseline period. The grey area represents the smallest worthwhile change of 0.5 x CV. *p < 0.05, **p < 0.01 compared to the preceding result. # = significant (*p* < 0.05) between-segment difference in the response. HR, heart rate; LnRMSSD, the natural logarithm of the root mean square of successive differences; SleepFull, average of the full sleep time; SleepEnd, end point of linear fit between 5-minute averages of full-night; SleepStart, starting point of linear fit between 5-minute averages of full-night data; Sleep4h, 4-hour period starting 30 min after going to sleep

The baseline vs. overload responses of nocturnal and morning HR and LnRMSSD are presented in Fig. 4. In turn, the correlations between the relative changes of different segments are presented in Table 2. Besides the results presented in Table 2, the relative change in supine and standing HR correlated significantly (*r* = 0.60, *p* = 0.002), while supine and standing LnRMSSD did not correlate significantly (*r* = 0.20, *p* = 0.347). The correlations between all nocturnal segments are presented in the Additional file 4.

### Associations to Fitness Level, Readiness, and Training Adaptations

**Fig. 5:**
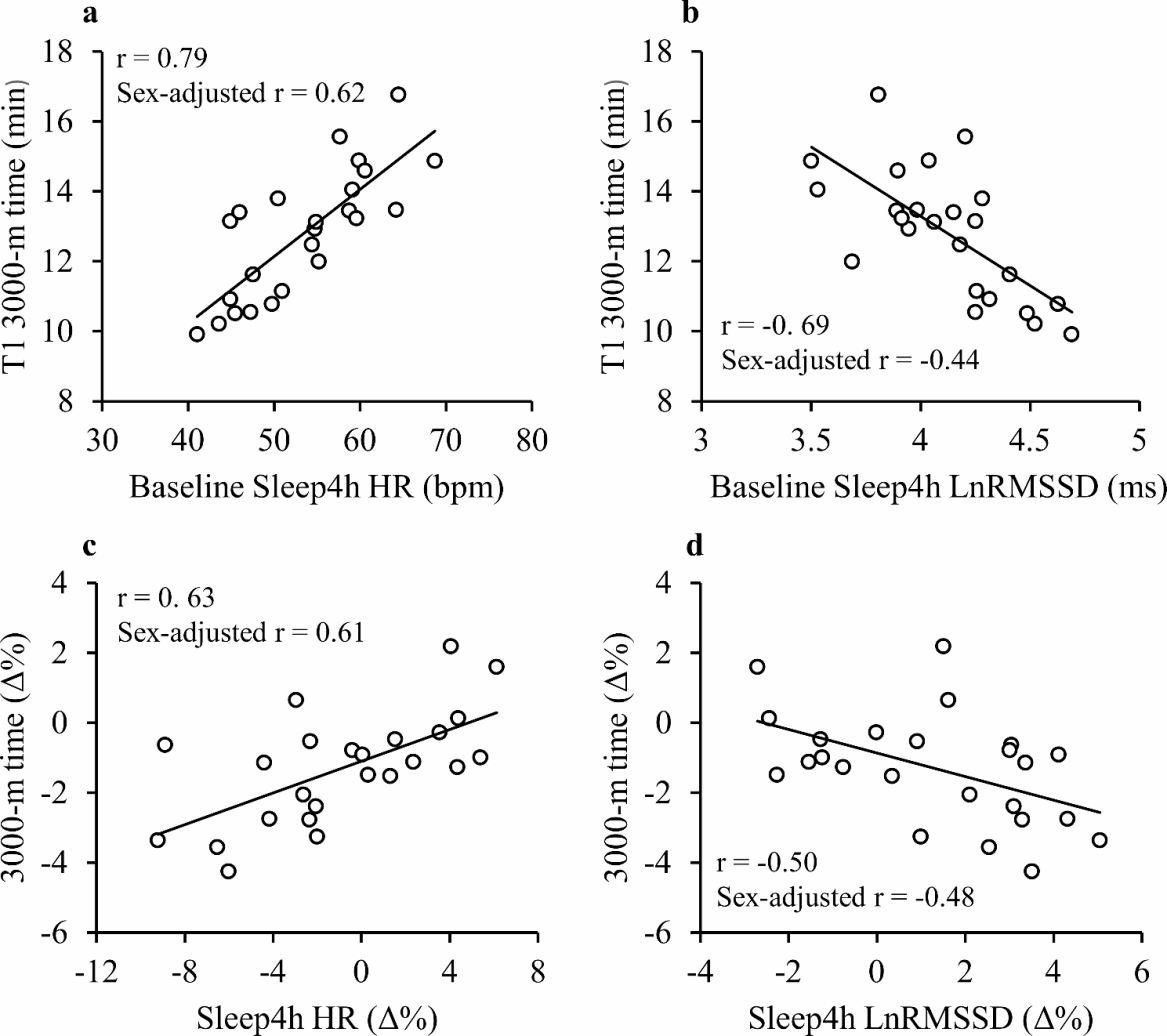
Correlations between the baseline 3000-m performance, nocturnal HR (**A**) and LnRMSSD (**B**), and their relative changes after the overload period (C = HR and D = LnRMSSD). The change in HR and LnRMSSD was analyzed from the mean of the baseline to the 7d-average at the end of the overload period. T1 refers to the time point after the baseline training period. Sleep4h, 4-hour period starting 30 min after going to sleep

The associations between baseline HR and LnRMSSD, baseline-overload responses, and 3000-m running performance are presented in Table 3. While morning and nocturnal HR segments correlated with the 3000-m performance at the baseline, the relative change in 3000-m performance correlated only with the relative changes of nocturnal segments of HR and Sleep4h LnRMSSD. The Pre-Post 3000-m change in HR or LnRMSSD was not associated with the baseline 3000-m performance or the change in performance after the overload period. Figure 5 demonstrates performance-related associations for the Sleep4h segment.

**Table 3:** Heart rate (HR) and LnRMSSD partial correlations (95% confidence intervals) with the baseline 3000-m time and the relative change in 3000-m time after the overload period. The correlations were adjusted for sex

	3000 m time T1 (min)	3000 m time (T1-T2 Δ%)
**HR (bpm)**		
*Morning supine BL* BL-OL Δ%	0.42* (-0.21;0.78) -0.13 (-0.52;0.33)	-0.344 (-0.70;0.18) 0.30 (-0.30;0.73)
*Morning standing BL* BL-OL Δ%	0.46* (0.00;0.82) 0.13 (-0.36;0.50)	-0.21 (-0.56;0.20) 0.22 (-0.43;0.69)
*SleepFull BL* BL-OL Δ%	0.61** (0.20;0.84) -0.03 (-0.51;0.48)	-0.41 (-0.76;0.08) 0.54** (0.18;0.78)
*Sleep4h BL* BL-OL Δ%	0.62** (0.26;0.84) -0.06 (-0.53;0.45)	-0.37 (-0.78;0.07) 0.61** (0.21;0.85)
*SleepStart BL* BL-OL Δ%	0.63** (0.29;0.84) -0.08 (-0.53;0.40)	-0.36 (-0.75;0.15) 0.54** (0.08;0.82)
*SleepEnd BL* BL-OL Δ%	0.52* (0.07;0.81) 0.06 (-0.34;0.52)	-0.42* (-0.72;0.00) 0.29 (-0.30;0.66)
**LnRMSSD (ms)**		
*Morning supine BL* BL-OL Δ%	-0.08 (-0.64;0.49) -0.06 (-0.50;0.33)	0.14 (-0.34;0.57) -0.10 (-0.53;0.43)
*Morning standing BL* BL-OL Δ%	-0.19 (-0.54;0.25) -0.21 (-0.58;0.20)	0.03 (-0.33;0.34) -0.08 (-0.45;0.38)
*SleepFull BL* BL-OL Δ%	-0.40 (-0.01;0.73) -0.22 (-0.69;0.29)	0.26 (-0.26;0.69) -0.38 (-0.69;-0.02)
*Sleep4h BL* BL-OL Δ%	-0.44* (-0.75;-0.06) -0.01 (-0.54;0.29)	0.28 (-0.22;0.70) -0.48* (-0.72;-0.17)
*SleepStart BL* BL-OL Δ%	-0.43* (-0.72;-0.04) -0.22 (-0.65;0.26)	0.23 (-0.32;0.68) -0.37 (-0.05;-0.66)
*SleepEnd BL* BL-OL Δ%	-0.33 (-0.70;0.07) -0.11 (-0.59;0.33)	0.25 (-0.20;0.64) -0.24 (-0.60;0.15)

BL, baseline training period; LnRMSSD, the natural logarithm of the root mean square of successive differences; OL, overload training period; SleepFull, average of the full sleep time; SleepEnd, an end point of linear fit between 5-minute averages of full-night; SleepStart, a starting point of linear fit between 5-minute averages of full-night data; Sleep4h, a 4-hour period starting 30 min after going to sleep.; T1, Time point after the baseline training period; T2, Time point after the overload period. *p < 0.05, **p < 0.01

In addition to the 3000-m running test, the associations between running performance and HR or LnRMSSD were analyzed with repeated measures correlation. The analyses were performed by pairing within-individual running speeds of all maximum sustainable effort interval sessions (6 sessions – 1/week) and the preceding sleep or morning HR and LnRMSSD result. The only significant correlations were found for Supine HR (*r* = -0.21, *p* = 0.014) and SleepEnd HR (*r* = -0.18, *p* = 0.049). All other correlations remained insignificant.

## Discussion

The results of the study demonstrated that although moderate to high correlations were found between morning and nocturnal HR and HRV in the long-term, the day-to-day or weekly responses to intensive training are not similarly aligned, especially in HRV. Furthermore, the nocturnal assessments seemed more responsive to maximal physical loading. Both recording conditions were associated with the baseline fitness level, but only the baseline-overload responses of nocturnal segments were associated with the change in the 3000-m running performance.

### Morning and Nocturnal Assessments at Baseline

In line with previous studies [26–28], most morning and nocturnal segments were at least moderately correlated at the baseline. As an exception, in the nocturnal segments of LnRMMSD there was low correlation with the standing LnRMSSD, while the supine and standing LnRMSSD had only negligible correlation. This highlights how the timing of the recording is not the only factor affecting the results, since different body positions (e.g. supine vs. standing) reflect different aspects of the autonomic nervous system modulation. In supine position, the HRV reflects primarily cardiac parasympathetic activity [16], but in the standing position during the orthostatic test, it reflects parasympathetic withdrawal and reactivation after a small stressor such as a change in the body position [35], and it is also affected by the sympathetic nervous system [39]. Apart from purely physiological aspects, one reason for the nocturnal LnRMSSD segments correlating more strongly with the standing position could relate to their smaller day-to-day variation (CV = 4.2–6.3%) compared with the supine position (CV = 8.2%). On the other hand, in the standing position, the CV was even twice (16.1%) the value of the supine position, which might reflect a challenge in terms of reliability. The sitting position was not measured in the current study, but it could potentially provide a more reliable option than the standing position to measure similar physiological aspects, as the absolute values seem to settle in between supine and standing positions [16], and the reproducibility has been comparable to the results of supine position in the present study [40].

### Responses to Maximum Exercise

The magnitude of HR and LnRMSSD responses after the 3000-m running test was the greatest in sleep segments (SleepStart, SleepFull, Sleep4h), while there were no significant changes in supine or standing HR and LnRMSSD. The greater response magnitude of sleep segments is, at least partially, affected by the fact that the running test was performed closer to the onset of sleep than the time when the orthostatic test was conducted (∼ 6 vs. ~14 h). In support of this, SleepEnd was the only sleep segment that did not change significantly after the test run. Contrasting the current supine and standing results, Stanley et al. [13] reported in their meta-analysis that it takes 24–48 h for HRV to recover after threshold-intensity exercise and at least 48 h after high-intensity training. On the other hand, more recent studies have not found significant changes in HRV 24 h after moderate- or high-intensity training sessions in awake recordings [19, 41] or during the night after high-intensity exercise [42] in recreational runners [19, 42] and highly trained rowers [41]. Therefore, it seems likely that systematic decreases in HRV require quite significant stimuli, such as maximum competition-like performance [20, 29], and in many individuals, HRV is restored within 24 h, at least in morning recordings. There were also significant interindividual differences in terms of the response magnitude and its direction, regardless of the recording condition or segment. This highlights potential differences in the disturbance of cardiovascular homeostasis that a single exercise induces. Although the acute recovery of HRV seems to be associated with the fitness level [13], the 3000-m time at the baseline did not correlate with any of the responses in the current study.

Somewhat surprisingly, the responses in sleep HR and LnRMSSD were more strongly associated (moderate correlations) with the responses of standing than supine position. This might suggest superior sensitivity of the standing position compared to the supine position in responses to previous day stressors. This is supported by the finding that the change in LnRMSSD in relation to individual CV was greater in the standing (-0.47) than in the supine position (-0.21). Hynynen et al. [43] have even suggested that standing results are more sensitive to perceived stress symptoms than nocturnal results. In nocturnal segments, the change in relation to individual CV was greater in HR (1.02 to 1.30) than in LnRMSSD (-0.70 to -0.87) in all other but SleepEnd (HR 0.04; LnRMSSD − 0.39) segments. This also aligns with the results of Thomas et al. [42] and Myllymäki et al. [44] who have found more significant changes in nocturnal HR compared to HRV in response to intensive exercise.

### Responses to Overload Period

While the 3000-m running test induced significant decrease in LnRMSSD and increase in HR, the responses to long-term intensive training seem to be more complex. In the current study, long-term responses were examined as the change from the baseline training period to the latter week of the overload period. As opposed to the acute responses, the LnRMSSD increased systematically in all segments except for SleepStart. In turn, the HR decreased significantly only in the standing position, and the magnitude of the changes was smaller than the ones in LnRMSSD. The results were in line with the studies of Le Meur et al. [45] and Bellinger et al. [46] who found increased HRV at the end of the overload period. The significant difference compared to their results was that in the current setting, the increased LnRMSSD was not associated with functional overreaching; instead, it actually reflected positive training adaptations. Although the meta-analysis of Manresa-Ramora et al. [21] has suggested that the ”parasympathetic hyperactivity” phenomenon (increase in HRV induced by high training load) would be associated with functional overreaching, this has not been observed among sleep recordings. Furthermore, as studies have found hyperactivity especially in the standing position [45, 46], it is possible that the phenomenon relates partly to diminished sympathetic activity that can occur in response to overload training [47].

All HR and LnRMSSD responses to the overload period demonstrated that even significant increases in the training load do not automatically alter the autonomic nervous system balance at rest. On the condition that the individual can tolerate the training load and keeps responding positively, it seems that the training is also effective. This is supported by the moderate correlations found with the nocturnal HR and LnRMSSD responses to the overload period and the changes in 3000-m running performance. However, as Herzig et al. [48] have suggested, when interpreting the HR and HRV responses, it might be challenging to separate the positive training adaptations (e.g., cardiac end diastolic volume) from the changes in the autonomic nervous system modulation. Endurance training interventions can also affect plasma volume [49], which can further have an impact on HR variables by itself [13]. Although current overload period can be considered quite short for significant cardiac adaptations, it cannot be stated decisively whether current responses were more due to training-induced adaptations or an outcome of a positive state of recovery and cardiac parasympathetic nervous system activity at the end of the overload period. However, the current results suggested that combining overload training with diminished parasympathetic nervous system activity would most likely lead to diminished training adaptations too.

Unlike in acute responses, the overload responses of nocturnal HR were more aligned with the results of supine than standing position. On the other hand, SleepEnd was the only segment that correlated with the standing response of LnRMSSD, and no such correlations were found in the supine position. This could reflect the slightly different factors affecting supine and standing recordings: standing can vary more in response to day-to-day stressors, while the responses of supine position are more stable, and might also relate more to endurance training induced adaptations.

### Is there an Optimal Timing for the Assessment of HR and HRV when Monitoring State of Recovery?

The purpose of longitudinal HRV monitoring in the context of sports is that it reflects the internal response to training and other stressors, rather than making comparisons against external training load metrics. Since the response is not similar across individuals despite a standardized load [19, 20], this information can further be applied into practice by considering whether an individual would be able to respond positively to new training stimuli or should the training load be adapted [22–24]. Although changes in HRV can align with the training adaptations [50–52], as they did also in the current study, the HRV should not be considered as a surrogate marker of maximal endurance capacity on a given day [53, 54]. The difference between “daily readiness” and chronic adaptations was partly demonstrated by the repeated measures correlations which found negligible correlations with the interval running performance and only for supine HR and SleepEnd HR. In practice, the optimal method for HRV monitoring should be considered in terms of how sensitively, validly, and reliably each method reflects meaningful changes in the general state of homeostasis.

The current study aimed at assessing the reliability of different HRV recording conditions and segments during a 3-week baseline period that was regarded as typical training. Accordingly, the assumption was that the CV results would represent day-to-day variability within each condition/segment, since the period was long enough for such an assessment, but not too long to increase the likelihood of significant adaptations or changes in the state of recovery. Importantly, the “noise” in the measurement itself does not yet inform whether a certain marker is useful. It is the magnitude of the “signal” in relation to noise (signal-to-noise-ratio) that defines the sensitivity of a marker, e.g., the change in HRV in response to stress [12].

The response to the 3000-m running test (acute) and the response to the overload period (chronic) could be regarded as signals in this study. Based on these results, the nocturnal segments seemed sensitive to respond into both directions: negatively to the 3000-m test but positively to the overload period that induced positive training adaptations, despite significant increases in volume and intensity of training. Furthermore, the magnitude of the changes in relation to the baseline CV was greater in nocturnal segments compared to morning assessments. An important side notion was that besides the recording condition, it should possibly be evaluated more critically whether HRV is significantly superior in its sensitivity compared to more simple HR assessments. In the current study, the responses were somewhat similar for the HR and LnRMSSD, and the associations with the training adaptations were even greater for the HR. This viewpoint has also been raised by Buchheit [12], and the question seems to remain quite relevant.

Regarding the limitations of morning and nocturnal assessments, both have had their own critiques. For example, some compliance-related challenges have been reported for morning [55] and nocturnal recordings [12]. Since then, new feasible and valid measurement technologies have been developed allowing shorter morning assessments without an HR strap [6] and more easily administered nocturnal assessments [4, 5]. Adherence to both conditions was very good in the current study (on average > 95%), suggesting no critical differences in terms of feasibility. Nocturnal assessments have also been criticized due to the sleep-stage dependency of HRV [56] and the distorting negative effect of the previous day’s training load on the results [12]. The current and previous studies [20, 26] suggest that potential day-to-day differences in sleep architecture do not affect the reproducibility of overnight measurements, at least when sufficiently long averaging periods are being used. Moreover, even intensive training in the evening does not necessarily seem to affect the HRV variables [42, 44, 57], and when such an effect has been observed, it has been associated with the subjective readiness to train on the following day [20]. One challenge in nocturnal recordings is that commercial devices such as watches [4] and rings [5] rely typically on PPG-based data. Although RR-interval and pulse-to-pulse-interval-based analyses have agreed well with several devices at rest [4–6, 8], it has also been argued that different methods are not unconditionally interchangeable [7]. PPG-based methods are also more prone to motion-induced artifacts [58], but these challenges are smaller during rest than during activities. Whatever the method being used is, the importance of appropriate artifact correction in HRV analysis should be acknowledged [59].

Finally, the morning assessments are always performed closer to the following exercise, and unlike nocturnal segments except for SleepEnd, it is affected by the physiological recovery occurring during sleep. Therefore, morning recordings could potentially represent better the actual readiness during the exercise. On the other hand, the larger day-to-day variation compared to sleep recordings is likely to reduce this advantage. When considering the method for the assessment of resting HR and HRV, the pros and cons of different methods should be critically evaluated. Further studies should address in more detail the physiological relevance and potential long-term consequences of suppressed parasympathetic nervous system activity during sleep vs. daytime.

### Limitations

The HR and HRV were monitored for six weeks, and it is not possible to extrapolate results directly to longer-term changes. The current setting focused on the training perspective, and the influence of other stressors cannot be estimated based on these findings. The recording methods were different for nocturnal (PPG) and morning assessments (HR-sensor), which may have affected especially the HRV comparisons. The artifact correction and signal filtering of the devices were based on proprietary algorithms that could not be adjusted by the research group. Despite the limitations, the study provided novel information regarding HR and HRV responses to intensified training and their associations to training adaptations with the devices that are widely used in the target group of recreational runners.

## Conclusions

Although morning and nocturnal HRV recordings provided relatively similar results in the long term, the responses to training were not similarly aligned. The nocturnal assessments responded significantly to acute physical stressor and were also associated with positive training-induced adaptations, supporting their usefulness in the monitoring of day-to-day and weekly responses to endurance training. Practitioners should not automatically assume that sleep and morning HR or HRV are exactly aligned in their responses. Future studies should investigate in more detail the potential long-term consequences of different types of HR and HRV patterns during sleep vs. waking hours.

## Electronic Supplementary Material

Below is the link to the electronic supplementary material.

Supplementary Material 1

Supplementary Material 2

Supplementary Material 3

Supplementary Material 4

## Data Availability

The datasets used and/or analyzed during the current study are available from the corresponding author on reasonable request.

## Abbreviations

BL: Baseline training period
CV: Coefficient of variation
HR: Heart rate
HRV: Heart rate variability
LnRMSSD: Natural logarithm of the root mean square of successive differences
OL: Overload training period
PPG: Photopletysmography
Sleep4h: 4-hour period starting 30 min after going to sleep
SleepEnd: End point of linear fit between 5-minute averages of full-night
SleepFull: Average of the full sleep time
SleepStart: Starting point of linear fit between 5-minute averages of full-night data
