# Correction: Morning versus Nocturnal Heart Rate and Heart Rate Variability Responses to Intensified Training in Recreational Runners

**DOI:** 10.1186/s40798-024-00806-5

**Published:** 2024-12-31

**Authors:** Olli-Pekka Nuuttila, Heikki Kyröläinen, Veli-Pekka Kokkonen, Arja Uusitalo

**Affiliations:** 1 Faculty of Sport and Health Sciences, University of Jyväskylä, P.O. Box 35(VIV), Jyväskylä, FIN-40014 Finland; 2 UKK Institute for Health Promotion Research, Tampere, Finland; 3 Department of Sports and Exercise Medicine, Clinicum, University of Helsinki, Helsinki, Finland; 4 Clinic for Sports and Exercise Medicine, Foundation for Sports and Exercise Medicine, Helsinki, Finland

**Sports Medicine - Open (2024) 10:120**

10.1186/s40798-024-00779-5

The original article contained an error whereby references 10–59 were mis-associated with their respective citations due to a minor typesetting error. This has since been amended.

