# Supplementary material for: Morning versus Nocturnal Heart Rate and Heart Rate Variability Responses to Intensified Training in Recreational Runners
Source: Sports Med Open. 2024 Nov 6;10:120. doi: 10.1186/s40798-024-00779-5 (PMC11541970; doi:10.1186/s40798-024-00779-5)
Supplement: Supplementary file 1 — Supplementary Material 1 [file 40798_2024_779_MOESM1_ESM.pdf]

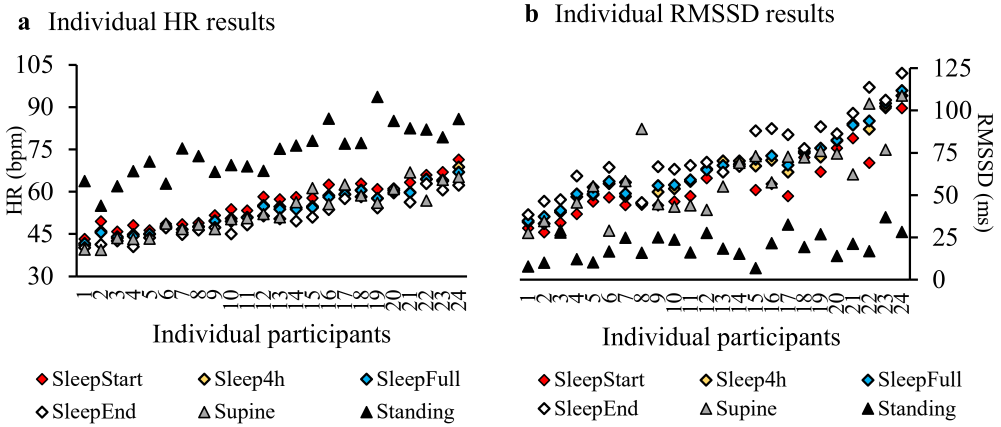

Additional file 1. Individual heart rate results (a) together with the absolute root mean square of successive differences results (b) at the baseline.
