# Supplementary material for: Morning versus Nocturnal Heart Rate and Heart Rate Variability Responses to Intensified Training in Recreational Runners
Source: Sports Med Open. 2024 Nov 6;10:120. doi: 10.1186/s40798-024-00779-5 (PMC11541970; doi:10.1186/s40798-024-00779-5)
Supplement: Supplementary file 2 — Supplementary Material 2 [file 40798_2024_779_MOESM2_ESM.pdf]

Morning versus nocturnal heart rate and heart rate variability responses to intensified training in recreational runners. Sports Medicine – Open. Olli-Pekka Nuuttila[1,2]*, Heikki Kyröläinen[1], Veli-Pekka Kokkonen[1], Arja Uusitalo[3,4]; 1 Faculty of Sport and Health Sciences, University of Jyväskylä, Finland; 2 UKK Institute for Health Promotion Research, Finland; 3 Department of Sports and Exercise Medicine, Clinicum, University of Helsinki, Finland; 4 Clinic for Sports and Exercise Medicine, Foundation for Sports and Exercise Medicine, Finland. *olli-pekka.s.nuuttila@jyu.fi,

Additional file 2 a. Pearson correlations and 95% confidence intervals (CI) between different recording segments at the baseline. Results of each segment are three-week averages.

Correlations

		HR Supine	HR Standing	HR SleepFull	HR Sleep4h	HR SleepStart	HR SleepEnd	LnRMSSD Supine	LnRMSSD Standing	LnRMSSD SleepFull	LnRMSSD Sleep4h	LnRMSSD SleepStart	LnRMSSD SleepEnd
HR Supine	Pearson Correlation	1	.780***	.914***	.909***	.902***	.899***	-.688***	-.481*	-.716***	-.721***	-.707***	-.667***
	95% CI		.550;.900	.810;.963	.797;.960	.794;.957	.778;.956	-.854;-.393	-.741;-.096	-.868;-.439	-.871;-.447	-.864;-.424	-.843;-.360
HR Standing	Pearson Correlation	.780***	1	.786***	.772***	.771***	.775***	-.301	-.704***	-.491*	-.508*	-.526**	-.427*
	95% CI	.550;.900		.560;.903	.535;.896	.533;.896	.540;.898	-.628;.117	-.862;-.420	-.746;-.109	-.756;-.131	-.767;-.156	-.708;-.029
HR SleepFull	Pearson Correlation	.914***	.786***	1	.997**	.985***	.982***	-.558**	-.437*	-.794***	-.809***	-.790***	-.735***
	95% CI	.810;.963	.560;.903		.992;.999	.965;.994	.958;.992	-..785;-.200	-.715;-.041	-.907;-.575	-.914;-.603	-.905;-.567	-.878;-.471
HR Sleep4h	Pearson Correlation	.909***	.772***	.997**	1	.993***	.966***	-.578**	-.414*	-.803***	-.822***	-.804***	-.738***
	95% CI	.797;.960	.535;.896	.992;.999		.984;.997	.923;.986	-.796;-.227	-.701;-.013	-.911;-.591	-.920;-.626	-.912;-.592	-.880;-.476
HR SleepStart	Pearson Correlation	.902***	.771***	.985***	.993***	1	.935***	-.591**	-.383	-.788***	-.812***	-.811***	-.704***
	95% CI	.794;.957	.533;.896	.965;.994	.984;.997		.853;.972	-.803;-.246	-.681;.025	-.904;-.563	-.916;-.608	-.915;-.607	-.862;-.419
HR SleepEnd	Pearson Correlation	.899***	.775***	.982***	.966***	.935***	1	-.509*	-.481*	-.774***	-.779***	-.739***	-.744***
	95% CI	.778;.956	.540;.898	.958;.992	.923;.986	.853;.972		-.757;-.133	-.741;-.096	-.897;-.540	-.900;-.547	-.880;-.478	-.882;-.486
LnRMSSD Supine	Pearson Correlation	-.688***	-.301	-.558**	-.578**	-.591**	-.509*	1	.223	.740***	.728***	.717***	.696***
	95% CI	-.854;-.393	-.628;.117	-..785;-.200	-.796;-.227	-.803;-.246	-.757;-.133		-.198;.575	.480;.881	.460;.875	.442;.869	.407;.858
LnRMSSD Standing	Pearson Correlation	-.481*	-.704***	-.437*	-.414*	-.383	-.481*	.223	1	.438*	.433*	.432*	.423*
	95% CI	-.741;-.096	-.862;-.420	-.715;-.041	-.701;-.013	-.681;.025	-.741;-.096	-.198;.575		.043;.715	.036;.712	.035;.712	.024;.706
LnRMSSD SleepFull	Pearson Correlation	-.716***	-.491*	-.794***	-.803***	-.788***	-.774***	.740***	.438*	1	.994***	.953***	.965***
	95% CI	-.868;-.439	-.746;-.109	-.907;-.575	-.911;-.591	-.904;-.563	-.897;-.540	.480;.881	.043;.715		.987;.998	.892;.980	.920;.985
LnRMSSD Sleep4h	Pearson Correlation	-.721***	-.508*	-.809***	-.822***	-.812***	-.779***	.728***	.433*	.994***	1	.974***	.936***
	95% CI	-.871;-.447	-.756;-.131	-.914;-.603	-.920;-.626	-.916;-.608	-.900;-.547	.460;.875	.036;.712	.987;.998		.940;.989	.856;.972
LnRMSSD SleepStart	Pearson Correlation	-.707***	-.526**	-.790***	-.804***	-.811***	-.739***	.717***	.432*	.953***	.974***	1	.841***
	95% CI	-.864;-.424	-.767;-.156	-.905;-.567	-.912;-.592	-.915;-.607	-.880;-.478	.442;.869	.035;.712	.892;.980	.940;.989		.662;.929
LnRMSSD SleepEnd	Pearson Correlation	-.667***	-.427*	-.735***	-.738***	-.704***	-.744***	.696***	.423*	.965***	.936***	.841***	1
	95% CI	-.843;-.360	-.708;-.029	-.878;-.471	-.880;-.476	-.862;-.419	-.882;-.486	.407;.858	.024;.706	.920;.985	.856;.972	.662;.929	

***p < 0.001, **p < 0.01, *p < 0.05. HR, heart rate; LnRMSSD, the natural logarithm of the root mean square of successive differences; SleepFull, average of the full sleep time; SleepEnd, end point of linear fit between 5-minute averages of full-night; SleepStart, starting point of linear fit between 5-minute averages of full-night data; Sleep4h, 4-hour period starting 30 minutes after going to sleep.

Additional file 2 b. Intraclass correlation coefficients (ICC) and 95% confidence intervals (CI) between different recording segments at the baseline. Results of each segment are three-week averages.

		HR Supine	HR Standing	HR SleepFull	HR Sleep4h	HR SleepStart	HR SleepEnd	LnRMSSD Supine	LnRMSSD Standing	LnRMSSD SleepFull	LnRMSSD Sleep4h	LnRMSSD SleepStart	LnRMSSD SleepEnd
HR Supine	ICC	1	.776***	.909***	.906***	.838***	.861***						
	95% CI		.548;.896	.804;.960	.797;.958	.374;.944	.628;.944						
HR Standing	ICC	.776***	1	.765***	.758***	.758***	.752***						
	95% CI	.548;.896		.530;.891	.516;.887	.518;.888	.507;.885						
HR SleepFull	ICC	.909***	.765***	1	.996***	.925***	.921***						
	95% CI	.804;.960	.530;.891		.991;.998	.029;.984	.030;.982						
HR Sleep4h	ICC	.906***	.758***	.996***	1	.942***	.901***						
	95% CI	.797;.958	.516;.887	.991;.998		.009;.988	.122;.975						
HR SleepStart	ICC	.838***	.758***	.925***	.942***	1	.744***						
	95% CI	.374;.944	.518;.888	.029;.984	.009;.988		.-068;.933						
HR SleepEnd	ICC	.861***	.752***	.921***	.901***	.744***	1						
	95% CI	.628;.944	.507;.885	.030;.982	.122;.975	.-068;.933							
LnRMSSD Supine	ICC							1	.221	.688***	.697***	.718***	.559***
	95% CI								-.192;.568	.387;.854	.420;.856	.449;.867	.070;.806
LnRMSSD Standing	ICC							.221	1	.412*	.410*	.419*	.395*
	95% CI							-.192;.568		.018;.694	.017;.694	.028;.699	.-0.001;.684
LnRMSSD SleepFull	ICC							.688***	.412*	1	.990***	.856***	.905***
	95% CI							.387;.854	.018;.694		.952;.997	.051;.962	.165;.976
LnRMSSD Sleep4h	ICC							.697***	.410*	.990***	1	.907***	.852***
	95% CI							.420;.856	.017;.694	.952;.997		.090;.977	.128;.958
LnRMSSD SleepStart	ICC							.718***	.419*	.856***	.907***	1	.629***
	95% CI							.449;.867	.028;.699	.051;.962	.090;.977		-.078;.878
LnRMSSD SleepEnd	ICC							.559***	.395*	.905***	.852***	.629***	1
	95% CI							.070;.806	.-0.001;.684	.165;.976	.128;.958	-.078;.878	

***p < 0.001, **p < 0.01, *p < 0.05. HR, heart rate; LnRMSSD, the natural logarithm of the root mean square of successive differences; SleepFull, average of the full sleep time; SleepEnd, end point of linear fit between 5-minute averages of full-night; SleepStart, starting point of linear fit between 5-minute averages of full-night data; Sleep4h, 4-hour period starting 30 minutes after going to sleep.
