# Supplementary material for: Morning versus Nocturnal Heart Rate and Heart Rate Variability Responses to Intensified Training in Recreational Runners
Source: Sports Med Open. 2024 Nov 6;10:120. doi: 10.1186/s40798-024-00779-5 (PMC11541970; doi:10.1186/s40798-024-00779-5)
Supplement: Supplementary file 3 — Supplementary Material 3 [file 40798_2024_779_MOESM3_ESM.pdf]

Morning versus nocturnal heart rate and heart rate variability responses to intensified training in recreational runners. Sports Medicine – Open. Olli-Pekka Nuuttila[1,2]*, Heikki Kyröläinen[1], Veli-Pekka Kokkonen[1], Arja Uusitalo[3,4]; 1 Faculty of Sport and Health Sciences, University of Jyväskylä, Finland; 2 UKK Institute for Health Promotion Research, Finland; 3 Department of Sports and Exercise Medicine, Clinicum, University of Helsinki, Finland; 4 Clinic for Sports and Exercise Medicine, Foundation for Sports and Exercise Medicine, Finland. *olli-pekka.s.nuuttila@jyu.fi.

Additional file 3. Pearson correlations and 95% confidence intervals (CI) between relative Pre-Post 3000-m changes of different recording segments. Change was analyzed from the recording preceding the 3000-m test day to the recording that followed the 3000-m test day.

Correlations

		HR Supine	HR Standing	HR SleepFull	HR Sleep4h	HR SleepStart	HR SleepEnd	LnRMSSD Supine	LnRMSSD Standing	LnRMSSD SleepFull	LnRMSSD Sleep4h	LnRMSSD SleepStart	LnRMSSD SleepEnd
HR Supine	Pearson Correlation	1	.394	.222	.175	.135	.346	-.478*	-.157	-.464*	-.397	-.309	-.347
	95% CI		-.012;.688	-.220;.589	-.266;.556	-.304;.527	-.089;.670	-.739;-.092	-.527;.263	-.741;-.052	-.801;.030	-.646;.130	-.671;.087
HR Standing	Pearson Correlation	.394	1	.673***	.684***	.716***	-.057	.129	-.583**	-.208	-.341	-.399	.124
	95% CI	-.012;.688		.350;.852	.368;.858	.421;.874	-.468;.373	-.289;.507	-.799;-.235	-.579;.234	-.667;.094	-.703;.027	-.314;.519
HR SleepFull	Pearson Correlation	.222	.673***	1	.975***	.957***	.299	.158	-.471*	-.433*	-.510*	-.592**	.025
	95% CI	-.220;.589	.350;.852		.939;.990	.897;.982	-.140;.640	-.282;.543	-.745;-.061	-.723;-.014	-.676;-.131	-.811;-.227	-.401;.442
HR Sleep4h	Pearson Correlation	.175	.684***	.975***	1	.952***	.223	.141	-.521*	-.411	-.524*	-.617**	.080
	95% CI	-.266;.556	.368;.858	.939;.990		.886;.980	-.219;.590	-.298;.531	-.773;-.127	-.710;.013	-.774;-.131	-.824;-.265	-.354;.485
HR SleepStart	Pearson Correlation	.135	.716***	.957***	.952***	1	.030	.283	-.531*	-.333	-.461*	-.597**	.169
	95% CI	-.304;.527	.421;.874	.897;.982	.886;.980		-.396;.446	-.157;.630	-.778;-.141	-.662;.103	-.739;-.049	-.814;-.234	-.272;.551
HR SleepEnd	Pearson Correlation	.346	-.057	.299	.223	.030	1	-.420	.148	-.364	-.157	-.007	-.480*
	95% CI	-.089;.670	-.468;.373	-.140;.640	-.219;.590	-.396;.446		-.715;.002	-.292;.536	-.681;.068	-.542;.284	-.427;.416	-.750;-.073
LnRMSSD Supine	Pearson Correlation	-.478*	.129	.223	.141	.283	-.420	1	.045	.196	.048	-.110	.426*
	95% CI	-.739;-.092	-.289;.507	-.282;.543	-.298;.531	-.157;.630	-.715;.002		-.365;.441	-.246;.571	-.382;.460	-.508;.326	.006;.719
LnRMSSD Standing	Pearson Correlation	-.157	-.583**	-.471*	-.521*	-.531*	.148	.045	1	.390	.533*	.535*	.033
	95% CI	-.527;.263	-.799;-.235	-.745;-.061	-.773;-.127	-.778;-.141	-.292;.536	-.365;.441		-.038;.697	.144;.779	.146;.780	-.394;.448
LnRMSSD SleepFull	Pearson Correlation	-.464*	-.208	-.433*	-.411	-.333	-.364	.196	.390	1	.883***	.760***	.687***
	95% CI	-.741;-.052	-.579;.234	-.723;-.014	-.710;.013	-.662;.103	-.681;.068	-.246;.571	-.038;.697		.735;.951	.498;.895	.373;.859
LnRMSSD Sleep4h	Pearson Correlation	-.397	-.341	-.510*	-.524*	-.461*	-.157	.048	.533*	.883***	1	.940***	.333
	95% CI	-.801;.030	-.667;.094	-.676;-.131	-.774;-.131	-.739;-.049	-.542;.284	-.382;.460	.144;.779	.735;.951	.	860;.975	-.103;.662
LnRMSSD SleepStart	Pearson Correlation	-.309	-.399	-.592**	-.617**	-.597**	-.007	-.110	.535*	.760***	.883***	1	.064
	95% CI	-.646;.130	-.703;.027	-.811;-.227	-.824;-.265	-.814;-.234	-.427;.416	-.508;.326	.146;.780	.498;.895	.735;.951		-.368;.473
LnRMSSD SleepEnd	Pearson Correlation	-.347	.124	.025	.080	.169	-.480*	.426*	.033	.687***	.333	.064	1
	95% CI	-.671;.087	-.314;.519	-.401;.442	-.354;.485	-.272;.551	-.750;-.073	.006;.719	-.394;.448	.373;.859	-.103;.662	-.368;.473	

***p < 0.001, **p < 0.01, *p < 0.05. HR, heart rate; LnRMSSD, the natural logarithm of the root mean square of successive differences; SleepFull, average of the full sleep time; SleepEnd, end point of linear fit between 5-minute averages of full-night; SleepStart, starting point of linear fit between 5-minute averages of full-night data; Sleep4h, 4-hour period starting 30 minutes after going to sleep.
