# Supplementary material for: Morning versus Nocturnal Heart Rate and Heart Rate Variability Responses to Intensified Training in Recreational Runners
Source: Sports Med Open. 2024 Nov 6;10:120. doi: 10.1186/s40798-024-00779-5 (PMC11541970; doi:10.1186/s40798-024-00779-5)
Supplement: Supplementary file 4 — Supplementary Material 4 [file 40798_2024_779_MOESM4_ESM.pdf]

Morning versus nocturnal heart rate and heart rate variability responses to intensified training in recreational runners. Sports Medicine – Open. Olli-Pekka Nuuttila[1,2]*, Heikki Kyröläinen[1], Veli-Pekka Kokkonen[1], Arja Uusitalo[3,4]; 1 Faculty of Sport and Health Sciences, University of Jyväskylä, Finland; 2 UKK Institute for Health Promotion Research, Finland; 3 Department of Sports and Exercise Medicine, Clinicum, University of Helsinki, Finland; 4 Clinic for Sports and Exercise Medicine, Foundation for Sports and Exercise Medicine, Finland. *olli-pekka.s.nuuttila@jyu.fi.

Additional file 4. Pearson correlations and 95% confidence intervals (CI) between relative baseline-overload changes of different recording segments. Change was analyzed from the average of the baseline period to the 7-d average at the end of the overload period.

Correlations													
		HR Supine	HR Standing	HR SleepFull	HR Sleep4h	HR SleepStart	HR SleepEnd	LnRMSSD Supine	LnRMSSD Standing	LnRMSSD SleepFull	LnRMSSD Sleep4h	LnRMSSD SleepStart	LnRMSSD SleepEnd
HR Supine	Pearson Correlation	1	.594**	.793***	.718***	.551**	.775***	-.558**	-.382	-.451*	-.437*	-.212	-.586**
	95% CI		.251;.805	.574;.907	.443;.870	.190;.781	.541;.898	-.785;-.200	-.680;.026	-.723;-.058	-.714;-.041	-.567;.209	-.800;-.239
HR Standing	Pearson Correlation	.594**	1	.535**	.480*	.367	.514*	-.503*	-.810***	-.195	-.168	-.007	-.387
	95% CI	.251;.805		.168;.772	.095;.740	-.043;.671	.140;.760	-.753;-.124	-.915;-.604	-.554;.227	-.535;.253	-.409;.398	-.684;.019
HR SleepFull	Pearson Correlation	.793***	.535**	1	.980***	.863***	.788***	-.346	-.379	-.703***	-.731***	-.588**	-.620**
	95% CI	.574;.907	.168;.772		.954;.992	.706;.939	.563;.904	-.658;.066	-.679;.029	-.862;-.418	-.876;-.465	-.801;-.242	-.819;-.289
HR Sleep4h	Pearson Correlation	.718***	.480*	.980***	1	.912***	.694***	-.219	-.345	-.712***	-.781***	-.657***	-.568**
	95% CI	.443;.870	.095;.740	.954;.992		.804;.961	.404;.857	-.572;.203	-.657;.068	-.867;-.434	-.901;-.551	-.838;-.345	-.790;-.213
HR SleepStart	Pearson Correlation	.551**	.367	.863***	.912***	1	.373	-.001	-.278	-.725***	-.806***	-.766***	-.477*
	95% CI	.190;.781	-.043;.671	.706;.939	.804;.961		-.036;.675	-.404;.403	-.613;141	-.873;-.454	-.913;-.596	-.894;-.526	-.739;-.092
HR SleepEnd	Pearson Correlation	.775***	.514*	.788***	.694***	.373	1	-.609**	-.340	-.416*	-.366	-.157	-.566**
	95% CI	.541;.898	.140;.760	.563;.904	.404;.857	-.036;.675		-.813;-.273	-.654;.073	-.702;-.015	-.671;.044	-.527;.263	-.789;-.211
LnRMSSD Supine	Pearson Correlation	-.558**	-.503*	-.346	-.219	-.001	-.609**	1	.201	.058	-.039	-.246	.310
	95% CI	-.785;-.200	-.753;-.124	-.658;.066	-.572;.203	-.404;.403	-.813;-.273		-.220;.559	-.354;.451	-.436;.370	-.591;.175	-.107;.634
LnRMSSD Standing	Pearson Correlation	-.382	-.810***	-.379	-.345	-.278	-.340	.201	1	.252	.128	.013	.454*
	95% CI	-.680;.026	-.915;-.604	-.679;.029	-.657;.068	-.613;141	-.654;.073	-.220;.559		-.168;.595	-.290;.505	-.392;.415	.062;.725
LnRMSSD SleepFull	Pearson Correlation	-.451*	-.195	-.703***	-.712***	-.725***	-.416*	.058	.252	1	.928***	.796***	.810***
	95% CI	-.723;-.058	-.554;.227	-.862;-.418	-.867;-.434	-.873;-.454	-.702;-.015	-.354;.451	-.168;.595		.838;.969	.579;.908	.603;.914
LnRMSSD Sleep4h	Pearson Correlation	-.437*	-.168	-.731***	-.781***	-.806***	-.366	-.039	.128	.928***	1	.877***	.641***
	95% CI	-.714;-.041	-.535;.253	-.876;-.465	-.901;-.551	-.913;-.596	-.671;.044	-.436;.370	-.290;.505	.838;.969		.733;.946	.320;.830
LnRMSSD SleepStart	Pearson Correlation	-.212	-.007	-.588**	-.657***	-.766***	-.157	-.246	.013	.796***	.877***	1	.314
	95% CI	-.567;.209	-.409;.398	-.801;-.242	-.838;-.345	-.894;-.526	-.527;.263	-.591;.175	-.392;.415	.579;.908	.733;.946		-.102;.637
LnRMSSD SleepEnd	Pearson Correlation	-.586**	-.387	-.620**	-.568**	-.477*	-.566**	.310	.454*	.810***	.641***	.314	1
	95% CI	-.800;-.239	-.684;.019	-.819;-.289	-.790;-.213	-.739;-.092	-.789;-.211	-.107;.634	.062;.725	.603;.914	.320;.830	-.102;.637	

***p < 0.001, **p < 0.01, *p < 0.05. HR, heart rate; LnRMSSD, the natural logarithm of the root mean square of successive differences; SleepFull, average of the full sleep time; SleepEnd, end point of linear fit between 5-minute averages of full-night; SleepStart, starting point of linear fit between 5-minute averages of full-night data; Sleep4h, 4-hour period starting 30 minutes after going to sleep.
